# Ferrous to Ferric Transition in Fe‐Phthalocyanine Driven by NO_2_ Exposure

**DOI:** 10.1002/chem.202004932

**Published:** 2021-01-25

**Authors:** Iulia Cojocariu, Silvia Carlotto, Henning Maximilian Sturmeit, Giovanni Zamborlini, Mirko Cinchetti, Albano Cossaro, Alberto Verdini, Luca Floreano, Matteo Jugovac, Peter Puschnig, Cinthia Piamonteze, Maurizio Casarin, Vitaliy Feyer, Claus Michael Schneider

**Affiliations:** ^1^ Peter Grünberg Institute (PGI-6) Forschungszentrum Jülich GmbH Leo-Brandt-Straße 52428 Jülich Germany; ^2^ Dipartimento di Scienze Chimiche Università degli Studi di Padova via F. Marzolo 1 35131 Padova Italy; ^3^ Technische Universität Dortmund Experimentelle Physik VI Otto-Hahn-Straße 4 44227 Dortmund Germany; ^4^ CNR-IOM Lab. TASC S.S. 14, Km. 163,5 34149 Trieste Italy; ^5^ Institute of Physics University of Graz, NAWI Graz Universitätsplatz 5 8010 Graz Austria; ^6^ Swiss Light Source Paul Scherrer Institute 5232 Villigen PSI Switzerland; ^7^ Fakultät für Physik and Center for Nanointegration Duisburg-Essen (CENIDE) Universität Duisburg-Essen Carl-Benz-Straße 199 47047 Duisburg Germany; ^8^ Present address: Istituto di Struttura della Materia-CNR (ISM-CNR) S.S. 14, Km. 163,5 34149 Trieste Italy

**Keywords:** molecular spintronics, oxidation state, phthalocyanine, surface science, XMCD

## Abstract

Due to its unique magnetic properties offered by the open‐shell electronic structure of the central metal ion, and for being an effective catalyst in a wide variety of reactions, iron phthalocyanine has drawn significant interest from the scientific community. Nevertheless, upon surface deposition, the magnetic properties of the molecular layer can be significantly affected by the coupling occurring at the interface, and the more reactive the surface, the stronger is the impact on the spin state. Here, we show that on Cu(100), indeed, the strong hybridization between the Fe d‐states of FePc and the sp‐band of the copper substrate modifies the charge distribution in the molecule, significantly influencing the magnetic properties of the iron ion. The Fe^II^ ion is stabilized in the low singlet spin state (*S*=0), leading to the complete quenching of the molecule magnetic moment. By exploiting the FePc/Cu(100) interface, we demonstrate that NO_2_ dissociation can be used to gradually change the magnetic properties of the iron ion, by trimming the gas dosage. For lower doses, the FePc film is decoupled from the copper substrate, restoring the gas phase triplet spin state (*S*=1). A higher dose induces the transition from ferrous to ferric phthalocyanine, in its intermediate spin state, with enhanced magnetic moment due to the interaction with the atomic ligands. Remarkably, in this way, three different spin configurations have been observed within the same metalorganic/metal interface by exposing it to different doses of NO_2_ at room temperature.

## Graphical abstract

**By exploiting the FePc/Cu(100)** interface, the possibility to control, via nitrogen dioxide (NO_2_) dissociation at the interface, the magnetic properties of the chelated ion the FePc array is demonstrated. Three different spin configurations have been stabilized within the same metalorganic/metal interface.

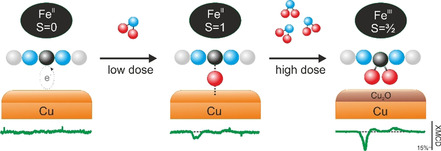

## Introduction

In the last decades, a great effort, both from the fundamental research and technological application point of view, has been made to exploit organic–metal interfaces in the engineering of functional molecular‐based spintronics devices.[^1^, ^2^, ^3^, ^4^] Among others, planar and aromatic metalorganic molecules, such as metal‐phthalocyanines (MPcs), metal‐porphyrins (MPs) and their derivatives, have been widely employed in molecular spintronics because they may manifest a broad variety of spin‐related phenomena, ranging from magnetic anisotropy[^5^, ^6^] to the Kondo effect.^[7]^ However, one of the main challenges is to preserve and enhance the magnetic properties of these complexes upon adsorption on a metal electrode while being able to precisely manipulate their spin states by external stimuli. In this context, the FePc molecules show a particular potential due to their unique magnetic properties as molecular magnets.[^8^, ^9^]

In general, upon adsorption on a metal surface, the iron center of a tetrapyrrolic compound can be subject to charge transfer, whose extent depends on both the nature of the chelated metal and the substrate reactivity. In particular, on coinage metals, the molecule‐substrate interaction is weaker when the FePc is deposited on gold; it increases on silver, reaching its maximum on copper. No changes in the electronic structure of the FePc occur upon interaction with Au,[^10^, ^11^] while in the case of Ag and Cu, the open Fe 3d shell hybridizes with the surface, leading to a charge transfer from the substrate to the molecular layer.[^10^, ^11^, ^12^] The coupling between the FePc and the substrate can alter or even completely quench the magnetic moment of the iron ion, because of the interaction occurring between the 3d shell and the substrate atoms.[^13^, ^14^, ^15^] In fact, the magnetic moment of the iron ion is retained upon the deposition of FePc on Au, whereas it almost vanishes when the molecule is deposited on Ag[^15^, ^16^] and it is completely quenched on Cu.^[12]^

The introduction of a buffer layer at the organic–metal interface can be exploited to tune the molecular‐surface interaction,[^17^, ^18^] even for restoring the gas phase triplet spin state of the iron ion together with its net magnetic moment. The latter has been achieved on the oxygen‐reconstructed copper surface, where the covalent nature of the Cu−O interaction yields a strong localization of the surface electrons inhibiting the charge transfer from the metal to the organic overlayer.[^17^, ^18^]

Metal‐phthalocyanines and metal‐porphyrins exhibit two possible sites for axially binding ligands to the central metal ion^[19]^ that can be used for spin manipulation, i.e., by changing the ligand field of the chelated ion.^[20]^ When these molecules are adsorbed on a surface, one of the two available binding sites is coordinated to the underlying substrate atom. The vacant site can be used both to influence the molecular‐surface interaction via the so‐called surface *trans*‐effect,[^21^, ^22^, ^23^, ^24^, ^25^] and to directly manipulate the spin and oxidation state of the ion within the tetrapyrrolic macrocycle. For instance, the local spin on the iron atom in FePc interfaced with the Au(111) substrate can be switched in a controlled way by ammonia and hydrogen adsorption.[^26^, ^27^] In some cases, the anchoring of atomic species to the metal ion in an organic array can switch‐on or enhance the magnetic moment in the metalorganic layer.[^16^, ^21^, ^28^]

**Scheme 1:**
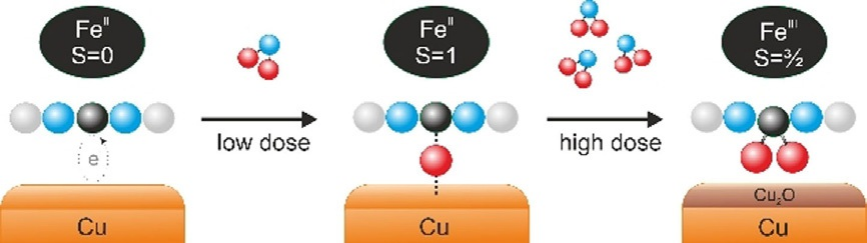
Schematic representation of the systems leading to the stabilization of three different spin configurations of the Fe center. Left: at the FePc/Cu(100) interface the Fe^II^ is stabilized in the singlet spin state due to the charge transfer from the copper substrate; middle: at low dose, the atomic oxygen generated from the NO_2_ dissociation at the interface partially decouples the molecule from the substrate stabilizing the Fe^II^ in the triplet spin state; right: after high NO_2_ dose the iron undergoes the ferrous to ferric transition due to the coordination of two oxygen atoms in *cis* position. Both at low and high NO_2_ doses the surface is involved in the oxidation process.

In the present work, we use a different strategy, which leads to the finely controlled modulation of the magnetic properties of the chelated ion the FePc array. By exploiting the FePc/Cu(100) interface, we demonstrate the possibility to restore, via nitrogen dioxide (NO_2_) dissociation at the interface, the magnetic moment of the metal ion of a Fe‐phthalocyanine molecule, which is quenched when the molecule is adsorbed on the copper (100) surface. Moreover, increasing the exposure of the FePc/Cu(100) interface to NO_2_ gas leads to the stabilization of a third spin configuration of the Fe ion (see Scheme 1).

By employing photoemission tomography, absorption, and photoemission spectroscopies, we show that a strong charge transfer takes place upon FePc adsorption on Cu(100), leading to a spin transition and the stabilization of the Fe ion in the low singlet spin state, totally quenching the magnetic moment of the organic layer. The gas‐phase triplet spin state of the Fe ion can be restored by exposure of the organic layer to intermediate doses of NO_2_. This process proceeds via NO_2_ dissociation at the interface and interaction of a single oxygen atom with the iron ion followed by the decoupling of the FePc molecules from the copper substrate. The exposure to higher doses of NO_2_ further changes the oxidation state of the central ion, and the ferrous to ferric transition is accompanied by a strong enhancement of the magnetic moment, as the iron oxidation increases the hole density. This corresponds to a final adsorption configuration where two oxygen atoms are interacting with the iron ion in *cis* position, forming the FePc(*η* ^2^‐O_2_) complex. Both the intermediate and final oxygen adsorption configurations are stable at room temperature.

The present results demonstrate explicitly that the magnetic moment of the iron ion can be manipulated by trimming the dose of the (Scheme 1) NO_2_ gas, which ultimately allows stabilizing three different spin configurations of the iron through the modification of its local oxidation state.

## Results and Discussion

### Quenching of the magnetic moment at the FePc/Cu(100) interface

Thanks to its relatively high reactivity, copper promotes a significant amount of charge to tetrapyrrolic compounds, as proved, for example, in the case of nickel‐containing porphyrins, where the lowest unoccupied molecular orbitals (LUMOs) are filled up to the LUMO+3^[29]^ and, at the same time, the molecule‐substrate interaction stabilizes the Ni ion in the uncommon +1 oxidation state.^[30]^

To study the energy level alignment of the FePc frontier orbitals upon interaction with copper, followed by a possible charge transfer caused by the molecule‐metal interaction, we performed photoemission tomography (PT) measurements combining momentum resolved photoemission experiments and theoretical calculations (see Photoemission Tomography simulations in Experimental Section).^[31]^

**Figure 1:**
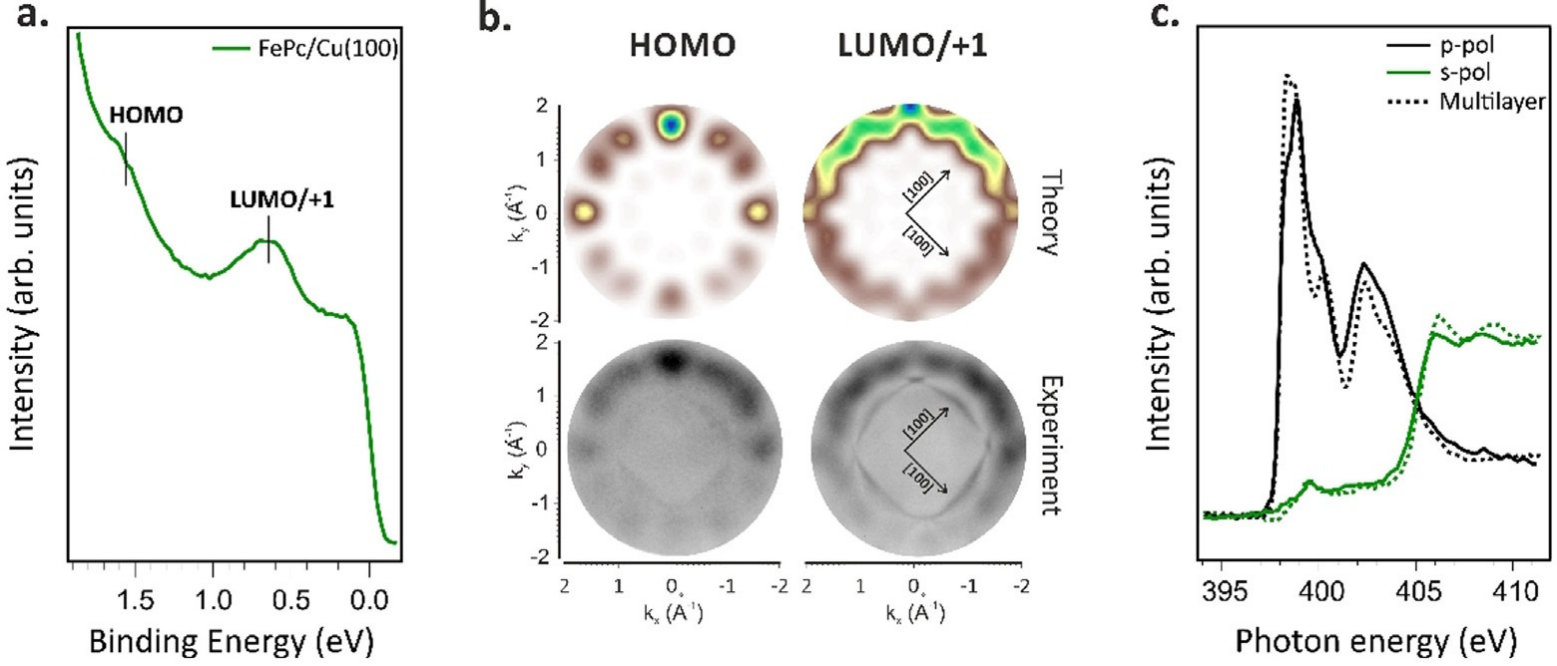
Valence band photoemission spectrum (a), and corresponding theoretical and experimental momentum maps (b) of a monolayer of FePc/Cu(100); N K‐edge absorption spectra acquired in the mono‐ and multilayer regime (c). Valence band spectrum and momentum maps acquired at 30 eV in *p*‐polarization.

Within the PT approach, the square modulus of the Fourier transform (FT) of the real space molecular orbitals can be directly related to the measured momentum distribution of the photoemitted electrons (momentum map) at defined binding energy (BE).[^29^, ^31^, ^32^, ^33^] As a result, this procedure allows to unequivocally assign an experimental valence band feature to a specific molecular frontier orbital. Figure 1 a shows the momentum integrated photoelectron spectrum of the FePc/Cu(100) interface measured at 30 eV using *p*‐polarized synchrotron radiation. While the valence band spectrum of the bare copper substrate shows a rather featureless plateau associated with sp‐bands,^[17]^ two prominent features are present in the FePc/Cu(100) spectrum, peaked at BEs 1.6 eV and 0.6 eV. To identify their origin, the momentum maps at corresponding BE were measured and the results are presented in the bottom row of Figure 1 b. The experimental pattern, in addition to the features originating from the molecular states, also contains sharp sp‐band contributions from the copper surface (visible at |**k**| ≈1.2 Å^−1^). The flat adsorption geometry of FePc, as determined from the N K‐edge spectrum (Figure 1 c), and the coexistence of two rotational domains due to the two‐fold symmetry of the Cu(100) substrate have been taken into account in the simulated maps (see Figure 1 b, top row). Furthermore, the intensity gradient along k_y_, deriving from the experimental geometry (25° incidence angle with respect to the surface), is well‐reproduced in the theoretical maps by including the |**A**⋅**k**| polarization factor.^[31]^ The FePc molecules result to be 29±5° mirrored with respect to the [100] high symmetry direction of the substrate, in reasonable agreement with the data reported in ref. [34]. Based on the excellent match between the experimental and theoretical data, the features at 1.6 eV and 0.6 eV BE, observed in the photoelectron spectrum of FePc/Cu(100) can be assigned to the emissions from the highest occupied molecular orbital (HOMO) of a_1u_ symmetry and the two degenerate LUMO/LUMO+1 of e_g_ symmetry of the FePc, respectively. Therefore, the occupation of the former LUMOs is associated with a strong molecular‐substrate interaction at the

FePc/Cu(100) interface and these orbitals are populated due to charge donation from the metal substrate to the molecular system. FePc has previously shown a similar behavior when deposited on Ag(100),^[35]^ but not on Au(100), suggesting a much weaker interaction of FePc with less reactive substrates and, as a consequence, no induced changes in the energy level alignment of the molecular electronic state upon adsorption.^[10]^

Complementary information on the charge transfer mechanism can be obtained by performing near‐edge X‐ray absorption fine structure (NEXAFS) spectroscopy.^[36]^ While PT is probing the occupied molecular states, NEXAFS is an efficient tool to study unoccupied and partially occupied molecular orbitals. The combination of these two techniques allows us to determine whether the molecular states are fully or partially filled by the charge transfer phenomenon occurring at the organic–metal interface. Changes in the electronic structure can be determined using NEXAFS by comparing the absorption spectra measured in the monolayer and multilayer regime. The latter is used as a reference for the gas‐phase like molecule and it is only weakly influenced by intermolecular and molecular‐substrate interactions. While, in the monolayer regime, the intensity and energy position of the absorption resonances is strongly influenced by the latter.

The NEXAFS spectra measured with *p*‐ and *s*‐polarized light across the N K‐edge, for both mono‐ and multilayer FePc deposited on Cu(100), are reported in Figure 1 c. The intense spectral features observed in the photon energy range of 397–404 eV in the multilayer spectrum measured using *p*‐polarization are assigned to the transition of N 1s electron to the π*‐symmetry unoccupied molecular orbitals, while the resonances above the 404 eV are attributed to the 1s→σ* transitions.^[37]^ The linear dichroism observed in the N K‐edge spectra for the FePc monolayer on Cu surfaces, i.e., the maximum intensity of the π* transitions in *p*‐polarization and the almost vanishing intensity of these resonances in the spectra measured in *s*‐polarization, indicates that the FePc molecules are highly oriented on the copper surface, with the molecular plane lying parallel to the substrate.^[38]^ Moreover, the decrease in intensity of the low energy π*‐symmetry resonances around 399 eV, in the FePc/Cu(100) monolayer spectrum (compared to the multilayer) supports the filling of the low energy LUMOs via the charge donation from the substrate to the adsorbed layer. This agrees with the PT measurements discussed previously (see Figure 1 a).

Having studied the charge transfer taking place at the interface, in the following, we discuss how the charge transfer influences the electronic and magnetic properties of the iron ion in the adsorbed FePc. To address this point, we performed XPS measurements of the Fe 2p_3/2_ core level, together with NEXAFS and XMCD experiments at the Fe l‐edge.

**Figure 2:**
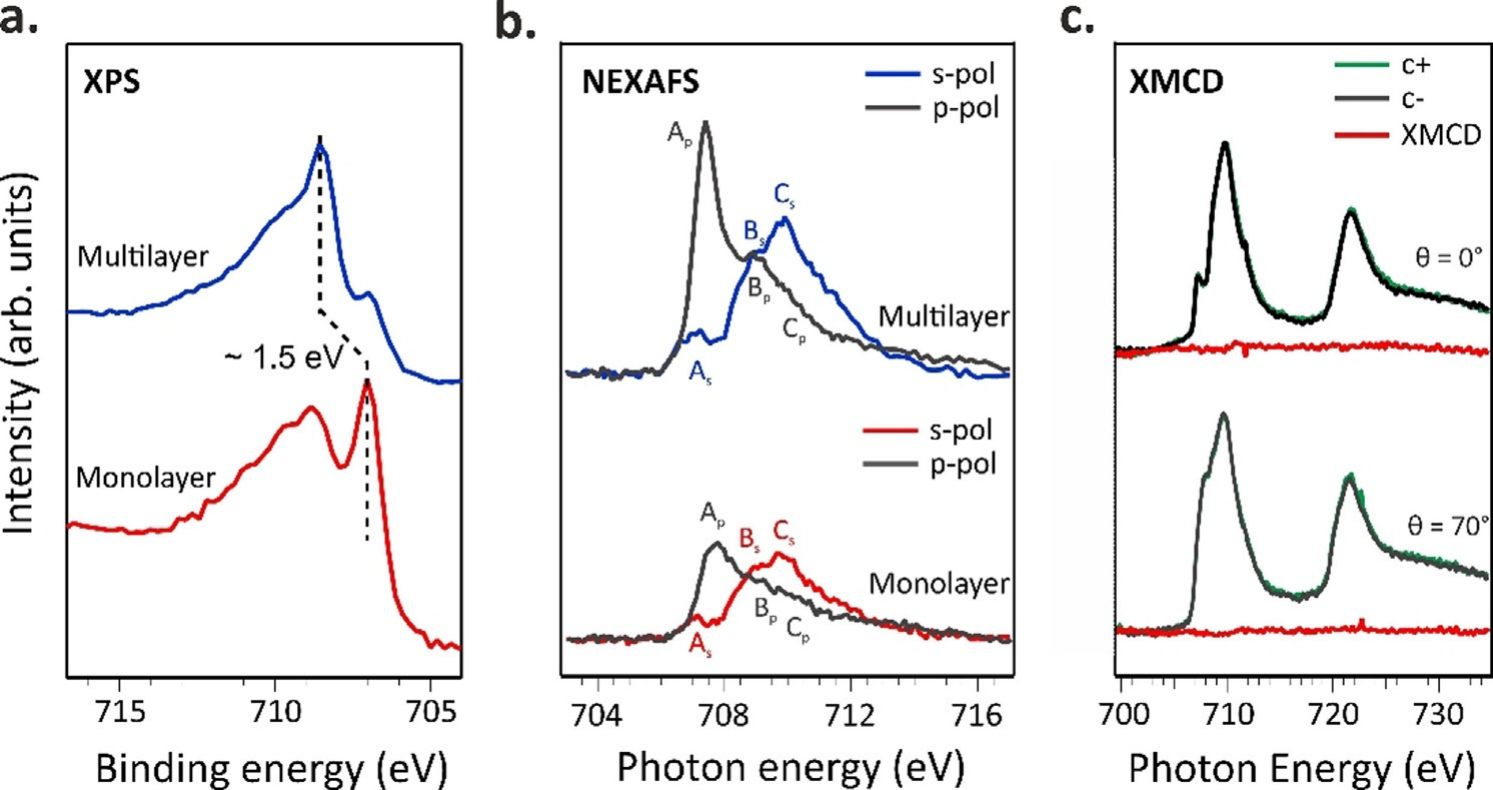
Fe 2p_3/2_ XPS (a) and Fe L_3_‐edge NEXAFS (b) spectra acquired at room temperature in the monolayer and multilayer regime; (c) Fe L_3,2_‐edge NEXAFS and XMCD measurements acquired at 3 K, while applying an external magnetic field of 4 T in the monolayer regime in grazing and normal incidence geometry. Fe XPS spectra (a) acquired at 910 eV.

In Figure 2 a, we compare the Fe 2p_3/2_ core level signals of FePc multilayer and monolayer adsorbed on the Cu(100) substrate. The monolayer spectrum, which consists of a mainline peaked at 707.05 eV and a high‐intensity satellite at higher BE (with the maximum at 708.8 eV), clearly resembles that of the FePc monolayer on Cu(110) substrate.^[12]^ The measurement of the Fe 2p_3/2_ core level of the multilayer phase (about 8 ML) displays a significant shift of the main peak to higher binding energies (≈1.5 eV) with respect to the monolayer case, as well as a notable change in the satellite features (see Figure 2 a). In the multilayer spectrum, a contribution from the first layer of FePc in direct contact with copper is still visible at 707.05 eV, suggesting that FePc deposition proceeds in a Stranski–Krastanov regime of growth.

Notably, the energy shift between mono‐ and multilayer of the Fe 2p_3/2_ mainline is much larger than the one observed for the peaks in the C 1s and N 1s spectra, namely 1.5 eV versus 0.1 eV (see Figure S1 in the Supporting Information). Both initial and final state effects may contribute to this large energy shift at the Fe 2p_3/2_. From XPS measurements alone, we cannot disentangle these two contributions to the chemical shifts and the change of the spectral shape of the satellite features in the monolayer and multilayer spectra.

To get direct access to the oxidation and spin state states of the Fe ion, we acquired absorption spectra for both multi‐ and monolayer FePc coverages at the Fe L_3_‐edge (Figure 2 b). Two groups of Fe  2p_3/2_ excitations with different polarization dependence are clearly visible from Figure 2 b: in *p*‐polarization, the features at 707.4 and 708.9 eV (features A_p_ and B_p_) are dominant in the spectra, whereas in *s*‐polarization the strongest peak C_s_ is observed at higher photon energy (709.8 eV). The Fe L_3_‐edge NEXAFS spectrum of the multilayer, as expected, resembles previously reported spectra for thin FePc film onto gold plated sapphire.^9^ The Fe L_3_‐edges NEXAFS data of the free FePc molecule was recently analyzed in great detail by Carlotto et al.^[39]^ Briefly, the 3d atomic orbitals of Fe ions transform as *a_1g_* (d${}_{z{}^{2}}$ )*, b_1g_* (d${}_{x{}^{2}\text{-}y{}^{2}}$ )*, b_2g_* (d_xy_) and *e_g_* (d_xz_, d_yz_) in a *D*_4*h*_ symmetry.

According to the DFT/ROCIS calculations (see Absorption spectra simulations in computational details), the electronic ground state (^3^E_g_) of free FePc corresponds to an intermediate state (IS) with a spin quantum number *S*=1 and a $a_{1g}^{1}b_{2g}^{1}e_{g}^{2}b_{1g}^{0}$ spin up (↑) and $b_{2g}^{1}e_{g}^{1}a_{1g}^{0}b_{1g}^{0}$ spin down (↓) electronic configuration. We would like to point out that, despite the unanimous consensus about the number of unpaired electrons (two) and spin state (*S*=1), different electronic terms and occupation numbers have been proposed in the literature.[^8^, ^9^, ^16^, ^19^, ^40^, ^41^, ^42^, ^43^, ^44^] According to our calculations, the lowest‐lying *A_p_* and *A_s_* features in the Fe L_3_‐edge multilayer spectra (see Figure 2 b) are both generated by Δ*S*=0 states associated with single Fe‐based 2p→3d electronic excitations involving the *a_1g_* and *e_g_* singly occupied MOs (SOMOs). Notably, the intensity of *A_p_* is significantly higher than that of *A_s_* clearly indicating that (a_2u_→a_1g_)^⊥^/(e_u_→e_g_)^⊥^ excitations give a stronger contribution to the spectra than the (a_2u_→e_g_)^∥^/(e_u_→a_1g_)^∥^ ones. Regarding the B and C features at 708.9 and 709.7 eV, respectively, DFT/ROCIS results allowed us to conclude that they are associated with both single and coupled‐single electronic excitations involving the 3d_xy_ virtual molecular orbitals (VMO).^[39]^

Comparing the multilayer to the monolayer spectra reported in Figure 2 b (bottom), we notice a reduction in the intensity of the low energy resonances *A_p_* and *A_s_*. Notably, the excitations at high photon energy have not shown such strong changes. By referring at the theoretical predictions reported in ref. [39], we can conclude that the transitions from the Fe 2p_3/2_ level to the *a_1g_* and *e_g_* molecular orbitals mainly contribute to the lower energy features in the spectra. The overall NEXAFS dichroism in the monolayer range is consistent with the expected out‐of‐plane‐oriented d${}_{z{}^{2}}$ *(a_1g_)* and d_xz_/d_yz_ (*e_g_*) molecular orbitals. These molecular orbitals have a stronger *z*‐direction component which is perpendicular to the copper surface, while the *b_1g_* (d${}_{x{}^{2}\text{-}y{}^{2}}$ )*, b_2g_* (d_xy_) orbitals lie mostly in the molecular plane parallel to the surface. Thus, the electrons of d${}_{z{}^{2}}$ and d_xz_/d_yz_ orbitals can better couple with the electrons in the substrate than those of *b_1g_* and *b_2g_* orbitals. Therefore, *a_1g_* and *e_g_* mainly participate in the molecule‐surface interaction and they are partially occupied due to the charge transfer between the copper substrate and the FePc molecules at the interface.

The rearrangement of the electronic states at the Fe center, upon interaction with the Cu surface, is also expected to influence the magnetic properties of the chelated ion.[^12^, ^15^, ^16^, ^45^] Thus, the magnetic configuration of the iron atom was probed using X‐ray magnetic circular dichroism (XMCD) measurements.

XMCD measurements were performed at 3 K while applying an external magnetic field of 4 T, which ensures the saturation of the magnetic moments in the FePc thin film regime.^[9]^ The magnetic moment of the Fe center ion is quenched on the Cu(100) surface, as evidenced by the absence of XMCD intensity both in in‐plane and in out‐of‐plane directions indicating that the total magnetic moment of Fe is null (±0.05 ${}_{\mu {}_{B}}$ ) (Figure 2 c), supporting the stabilization of the Fe^II^ singlet state (*S*=0), in agreement with ref. [12]. We suggest that this could be due to the enhanced coupling of the Fe d‐states with the *sp*‐band of copper substrate electrons. This is in clear contrast with the FePc multilayer^[9]^ and FePc adsorbed on Au(111),^[15]^ where an XMCD signal is clearly visible. The change of the spin state in the adsorbed FePc molecule on a bare copper surface is also associated with the changes in energy position and shape of the Fe 2p_3/2_ core‐level spectra discussed above.

### Enhancement of the magnetic moment by external chemical stimuli

**Figure 3:**
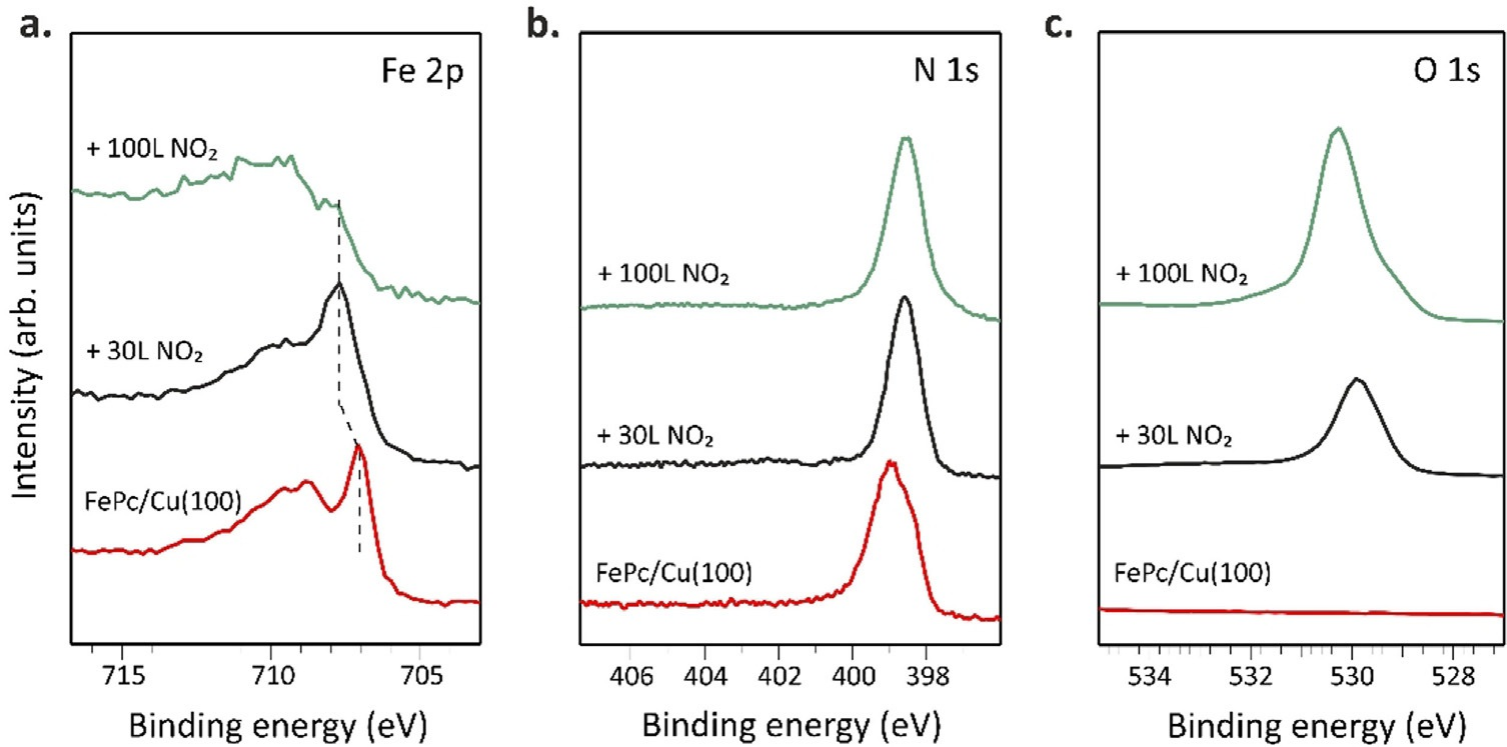
XPS spectra of FePc deposited on Cu(100) before and after exposure to increasing NO_2_ doses acquired at the Fe 2p_3/2_ (a) and N 1s (b) and O 1s (c) level. XPS spectra acquired at 910, 515 and 650 eV, respectively.

To restore the magnetic moment of the ion, which is quenched due to the molecule‐surface interaction, two different approaches were previously proposed: electron doping[^16^, ^46^] or functionalization with an external ligand, e.g., a small gaseous molecule.[^21^, ^22^, ^23^, ^24^, ^25^] Inspired by the latter, we exposed the FePc/Cu(100) interface to gaseous NO_2_ to restore the magnetic moment of the iron ion. The interface was exposed to two different NO_2_ doses, 30 and 100 L (referred to as low and high dose, respectively, throughout the text), and the changes at the Fe ion were followed by measuring the Fe 2p_3/2_ core‐level at increasing NO_2_ exposure (see Figure 3 a). After the low dose, the Fe 2p_3/2_ spectrum resembles the one of the multilayer, showing the characteristics Fe^II^ structures (see for comparison Figure 2 a), i.e., a sharp peak at lower and broad satellite features at higher BE. However, after the high dose, we witness a quenching of the sharp line (see Figure 3 a, top).

At this point, two different scenarios regarding the interaction of NO_2_ with the Fe ion have to be considered. 1) The NO_2_ molecules bind to the Fe center of surface‐anchored phthalocyanines in *trans* position, decreasing the strength of the molecule‐surface interaction via the *trans*‐effect.^[19]^ 2) The NO_2_ molecules dissociate at the FePc/Cu(100) interface. In the latter scenario, the corresponding products can bind to the molecule in the *trans* position or intercalate between the substrate and the molecular overlayer. Further considerations on the dissociation at the interface will be discussed in the following.

The absence of a new component (expected to rise at higher binding energies) in the N 1s spectra measured after both low and high dose of NO_2_ (Figure 3 b), as well as the conservation of the area below the peak related to the Pc nitrogen atoms, excludes the first scenario, in which intact NO_2_ or other nitrogen‐containing products bind to the coordinated iron atom. Therefore, the changes in the iron core‐level spectra are likely caused by the oxygen atoms created at the interface by an on‐surface reaction involving the dissociation of NO_2_ molecules (the presence of oxygen at the FePc/Cu(100) interface is confirmed by the O 1s spectra reported in Figure 3 c). To elucidate whether oxygen atoms are anchored on top of the molecular layer (in *trans* position) or placed between the molecular layer and the copper substrate, the FePc molecules have been sublimated on an oxygen pre‐exposed copper surface (O−Cu(100)), which shows a (√2×√2)R45° reconstruction.^[48]^ The Fe 2p_3/2_, O 1s, N 1s, C 1s and Cu 3p spectra of FePc/O−Cu(100) interface are shown in Figure S3, and they very well resemble the corresponding spectra of FePc/Cu(100) after the exposure to the low NO_2_ dose (see Figure 3 a), supporting a similar chemical environment of iron in the two systems. This suggests that, upon the low NO_2_ dose, oxygen atoms are formed after a dissociation reaction at the FePc/Cu(100) interface and are chemisorbed on the copper surface. This is well evident in the O 1s spectra, where both core level spectra of O−Cu(100) and FePc/Cu(100) after low dose are characterized by a similar BE (530.0 eV) and line shape, a fingerprint of chemisorbed oxygen atoms on the copper surface. Instead, after dosing 100 L of NO_2_ the O 1s spectrum shows a clear chemical shift of the main feature to higher BE (530.3 eV) as well as the shoulder at lower BE (529.1 eV); associated with the oxidation of the copper substrate underneath the molecular layer (Cu_2_O and CuO, respectively).^[51]^

Besides, the linear dichroism observed in the O K‐edge NEXAFS spectra also confirms the presence of atomic oxygen chemisorbed on the copper surface, without further oxidation of the copper substrate. However, the clear changes in spectral shape and energy position in the O K‐edge NEXAFS spectrum of FePc/O−Cu(100) compared to the bare O−Cu(100) substrate spectrum (see Figure S4) suggest that FePc molecules are not fully decoupled electronically and physically from the chemisorbed oxygen, neither for FePc/O−Cu(100) nor the FePc/Cu(100) interface exposed to the low NO_2_ dose.

**Figure 4:**
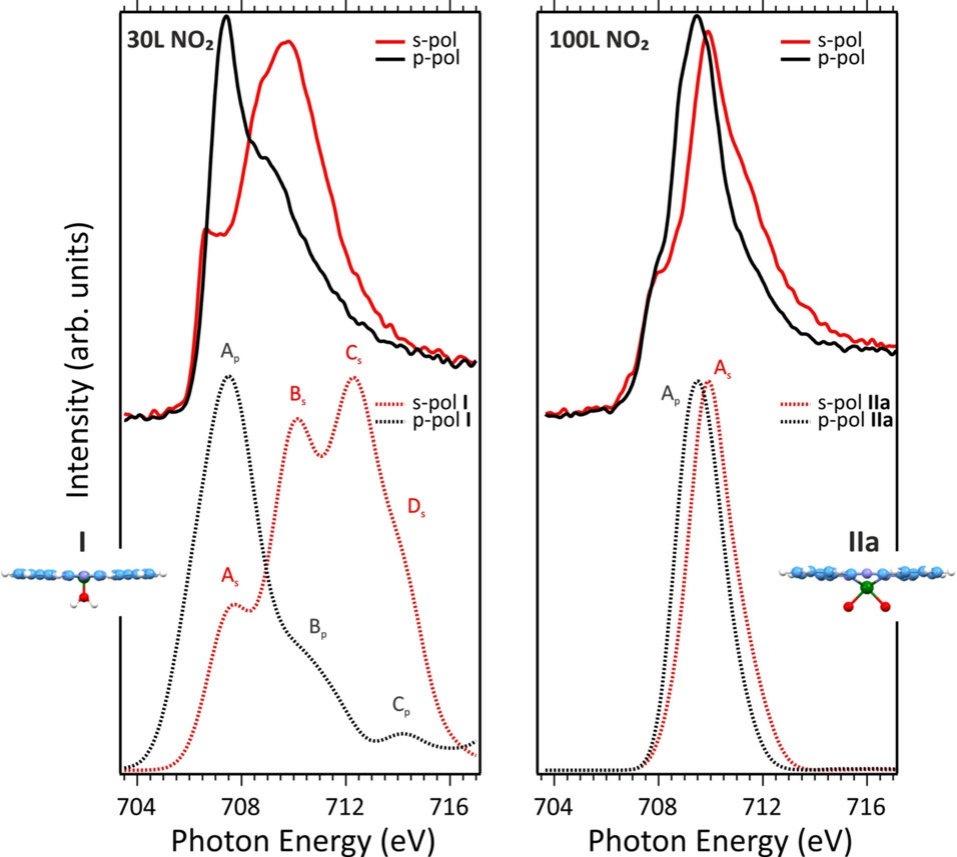
Top row: NEXAFS spectra of FePc/Cu(100) exposed to 30 L of NO_2_ (left) and to 100 L of NO_2_ (right). Bottom row: Fe L_3_‐edge NEXAFS spectra simulated for the *s*‐ and *p*‐polarized excitation. Normalized simulated spectra for **I** and **II a** are shifted of 13.4 and 14.5 eV, respectively and have a Gaussian broadening of 2 and 1.3 eV, respectively; corresponding optimized structures for **I** and **II a**. Blue, white, violet, red and green spheres are C, H, N, O and Fe atoms.

To gain further insights into the low and high NO_2_ dose trends, especially about the oxygen atoms coordination to the Fe ion, we simulated different structural arrangements for FePc on Cu(100). It is noteworthy that a leading role in determining the Fe L_3_‐edge NEXAFS spectrum^[39]^ is played by the Fe nearest neighbors; thus, for the low NO_2_ dose, the nature of the weakly interacting oxygen with FePc determined from O K‐edge NEXAFS data is critical, while the character of the O−Cu interaction is less relevant. The adoption of the molecular cluster approach to model a periodic system implies the saturation of the oxygen dangling bonds with hydrogen/pseudo‐hydrogen atoms.^[49]^ Therefore, the coordinated system resulting from low NO_2_ dosing has been modelled by considering the free molecular complex **I** (see its optimized structure in Figure 4) characterized by the presence of a single oxygen atom of water molecule placed at 1.8 Å from the Fe^II^ ion.^[50]^ The Fe L‐edge NEXAFS modelling has been carried out for both *s*‐ and *p*‐polarized excitation. The final good agreement between theory and experiment is a clear indication of the adopted model feasibility.

The comparison between theoretical results (see Figure 4, bottom row) and experimental evidence recorded at low (30 L) NO_2_ dosing (see Figure 4, top row) encourages us to assess that NO_2_ initially dissociates at the FePc/Cu(100) interface and generates single oxygen atoms, which intercalate between the FePc layer and chemisorbs on the copper surface. The intercalation of the oxygen atoms results in the partial decoupling of the molecules from the substrate and the restoring the original FePc gas‐phase spin state (*S*=1). As far as the detailed assignment of the L_3_‐edge spectrum of the decoupled FePc is concerned, the lowest‐lying feature in *p* (*A_p_*) and *s* (*A_s_*) polarization (see Figure 4, bottom row) are both associated with electronic states with Δ*S*=0, generated by single Fe 2p→Fe 3d electronic excitations involving the 3d${}_{z{}^{2}}$ and 3d_xz_ singly occupied MOs (SOMOs). In contrast, Δ*S*=0, ±1 electronic states contribute to B_s_. Single (2p→d${}_{z{}^{2}}$ /π^*^ Pc‐based VMO) and coupled‐single (Fe 2p→3d_xz_ and 3d_xz_→3d_xy_/π^*^ Pc‐based VMOs) electronic excitations generate Δ*S*=0 states, while the Δ*S*=±1 states are all associated to metal‐to‐ligand‐charge‐transfer (MLCT) single electronic transitions. Only electronic states with Δ*S*=0, −1 contribute to *B_p_*; both of them imply single electronic transitions, the former states have a Fe 2p→3d SOMOs nature; the latter ones, analogously to *B_s_*, display an MLCT character. Finally, only Δ*S*=0 electronic states contribute to *C_s_* and *D_s_* through single and coupled‐single excitations having once again an MLCT character.

To get information about the most favorable adsorption structure formed after exposing FePc/Cu(100) to high NO_2_ doses, we have examined different geometries considering two possible Fe oxidation states, i.e., Fe^III^ and Fe^IV^. The presence of the former has been modelled by considering two O atoms coordinated to FePc with a pseudo‐peroxide coordination, the FePc(*η* ^2^‐O_2_) complex, (**II a**, see Figure 4) whose electronic properties have been thoroughly described in ref. [39]. As far as Fe^IV^ is concerned, two different models have been tested: the former implied the presence of two O atoms in a *trans* arrangement (**II b,** Figure S2) with respect to FePc plane, while the latter involves the formation of an oxoiron(IV)Pc (**II c**) (Figure S2) complex. Among the different spin states considered for **II b** and **II c**, the most stable species correspond to a triplet spin state with two unpaired electrons on the Fe=O fragment in agreement with the literature.^[53]^ As such, it is noteworthy that linear dichroism is well evident in the modeled spectra of **II b** and **II c** (see Figure S2), while it is absent in the spectra of **II a** complex (see Figure 4). As a whole, the comparison between simulated and experimental NEXAFS spectra at high (100 L) NO_2_ dosage shows that **II a** is the most favorable complex formed at the interface and rules out the presence of a relevant percentage of **II b** or **II c** species.

A similar scenario has been observed for the FePc/Ag(110) interface exposed to oxygen.[^39^, ^47^] The use of the FePc(*η* ^2^‐O_2_) cluster shows a very good agreement between theory and experiment thus providing support to the presence of oxygen atoms lying in between FePc and the substrate and information about the relevant role of the substrate on the NO_2_ dissociation. In detail, the single peak characterizing both the *s*‐ and *p*‐polarized L_3_‐edge spectrum of the *cis* complex is mainly (80 %) due to electronic states associated to transitions having Δ*S*=0 and corresponding to Fe^III^ 2p‐based→3d‐based single electronic excitations involving the d${}_{z{}^{2}}$ , d_xz_ and d_xy_ SOMOs. Interestingly, the MLCT electronic state generated by Fe^III^ 2p→π* MLCT excitations with Δ*S*=−1 significantly contribute to the higher excitation energy side of both peaks. Despite the overall agreement between experimental and simulated (see Figure 4, right) spectra, we have to point out that in the simulated spectrum **II a** the evident shoulder on the lower excitation energy side of the *s*‐ and *p*‐polarized spectra is not well reproduced. This feature is rather associated with the co‐presence at the interface of residual molecules in the single‐oxygen structure **I**.

The NEXAFS spectra at O K‐edge measured after the high dose (see Figure S4) are in good agreement with previous measurements for Cu_2_O,[^51^, ^52^] thus supporting the oxidation of the copper substrate after the exposure of FePc/Cu(100) interface to high NO_2_ dose.

To summarize, the calculation shows that the **I** structure (low NO_2_ dose) is associated with Fe^II^ species with an intermediate spin state (*S*=1), while binding of the second oxygen atom (**II a** configuration) induces the oxidation of the iron ion (Fe^II^→Fe^III^ transition). In agreement with theoretical and experimental data, the *trans* adsorption configuration (**II b** structure) is not formed at the present experimental condition.

**Figure 5:**
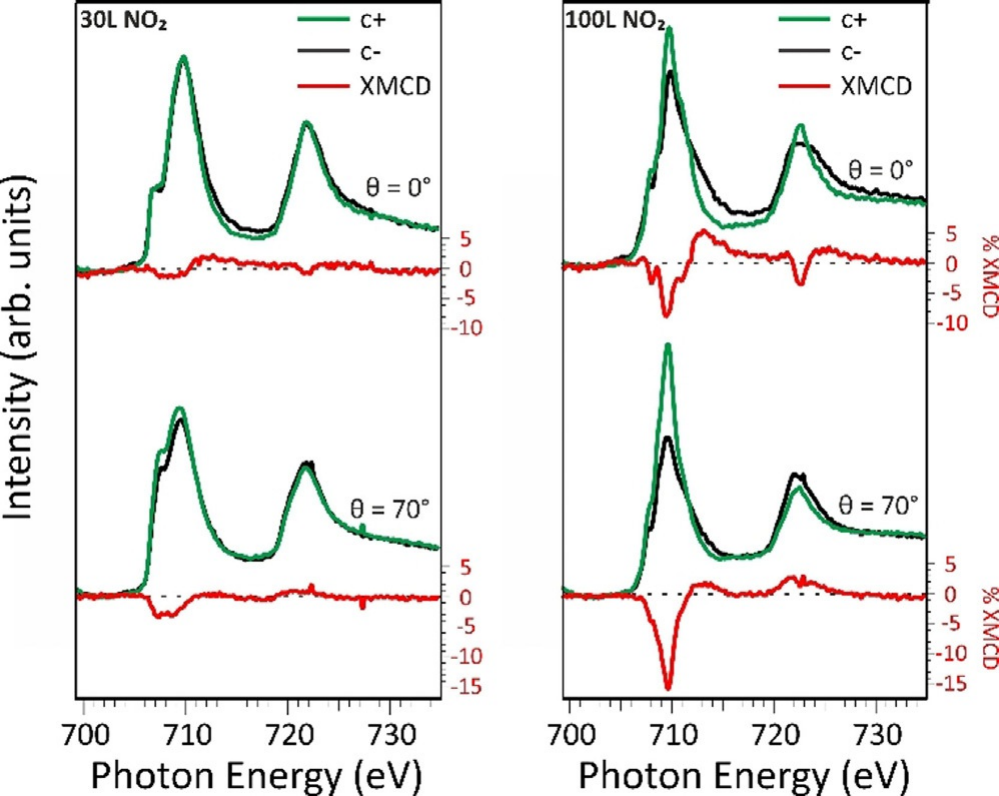
NEXAFS and XMCD spectra of FePc/Cu(100) exposed to 30 L of NO_2_ (left) and to 100 L of NO_2_ (right), acquired at 3 K, while applying an external magnetic field of 4 T.

At this point, we can analyze the changes of the spin states and oxidation states in the **I** and **II a** structures in comparison with XMCD data measured at Fe L_3,2_‐edge after stepwise increasing the dose of NO_2_ (see Figure 5).

As previously remarked, the Fe l‐edge XMCD of the FePc is completely quenched on Cu(100). However, exposing the system to increasing NO_2_ doses, the iron magnetic moment gradually increases and develops a sizeable in‐plane magnetic anisotropy. The orbital and spin components of the Fe magnetic moment projected along the field direction for a given incidence angle can be obtained from the sum rule analysis.[^54^, ^55^] It has to be noticed that in the effective spin magnetic moment obtained from the sum rule analysis, the dipolar term T_z_ could induce big discrepancies between the effective m${}_{S{}_{\mathrm{eff}}}$ and m_S_. To take account of this, based on previous multiplet calculations on the FePc system, we considered an error of 30 % on m_S_.^[56]^ For calculating the magnetic moments, we have assumed 4 holes h_d_ for the intermediate dose and 5 holes for the higher dose, with the holes localized on the SOMO d${}_{z{}^{2}}$ , d_xy_ and d_xz_ and on the completely empty d_yz_.^[39]^ The XMCD measurements for the lower NO_2_ dose confirm the recovery of the triplet spin state (*S*=1). Indeed, the total magnetic moment m_tot_ in the molecular plane (0.46 μ_B_, m_S_ and m_L_ values for the two doses are given in Table S6 for the in‐plane direction) is comparable with the one reported for a film of 0.5 ML FePc deposited on a ferromagnetic Co(001) substrate (0.56 μ_B_).^[14]^ Interestingly, the molecule‐surface interaction on copper, hence the charge transfer, appears to be stronger than on cobalt, where the Fe magnetic moment is preserved. Comparing the intermediate and high dose XMCD spectra, a strong increase of the m_tot_ to 2.14 μ_B_ (see Table S6) further confirms the change in the oxidation state, as the transition from ferrous to ferric phthalocyanine is followed by the increasing of the number of unpaired electrons (from 2 to 3) that contribute to the organic layer magnetism.

As the XMCD intensity is proportional to the projection of the magnetic moment along the X‐ray incidence direction, the higher intensity of the measured XMCD at grazing (*θ*=70°) rather than normal (*θ*=0°) incidence leads us to conclude that the system exhibits a preferential in‐plane magnetic anisotropy at both low and high NO_2_ dosing. The observed changes in the magnetic anisotropy induced by oxygen coordination are consistent with previous studies.[^9^, ^28^]

## Conclusion

By combining the PT, XPS and NEXAFS experimental techniques along with theoretical simulations, we have shown that the controlled modulation of the spin state of a metalorganic network can be achieved by coordination of the chelated ion with small ligands that modify the molecule–substrate interaction. While the copper surface quenches the magnetic moment of the metal ion, the exposure to NO_2_ and, consequently, the coordination with atomic oxygen, formed due to the NO_2_ dissociation at the interface, gradually modifies the magnitude and orientation of the magnetization. For low NO_2_ doses, FePc is decoupled from the copper substrate by the intercalation of atomic oxygen and the molecular network recovers the typical gas phase magnetic moment. In this regime, a single oxygen atom binds to the iron ion weakening the hybridization and the charge transfer effect at the interface, the two phenomena which are responsible for the quenching of the magnetic moment at the FePc/Cu(100) interface. With increasing NO_2_ doses, the central iron ion interacts with two oxygen atoms in a FePc(*η* ^2^‐O_2_) configuration, both transferred to the surface, and all the coordinated sites undergo the ferrous to ferric transition (from Fe^II^ to Fe^III^), with a strong increase of the magnetic moment and the in‐plane anisotropy. The FePc(*η* ^2^‐O_2_) complex, where two O atoms are coordinated to the Fe ion in a pseudo‐peroxide geometry, shows to be most favorable from among of the different final structures considered.

## Experimental Section

**Methods and equipment**: The valence band photoemission spectra were measured at the NanoESCA beamline of Elettra, the Italian synchrotron radiation facility in Trieste, using an electrostatic photoemission electron microscope (PEEM) set‐up described in detail in ref. [57]. The data were collected with a photon energy of 30 eV and a total energy resolution of 100 meV, using *p*‐linearly polarized light. The NEXAFS measurements of bare FePc/Cu(100) interface were performed at the ALOISA beamline, also located at Elettra synchrotron.^[58]^ The spectra across C, N and O K‐edge were taken in partial electron yield mode using a Channeltron multiplier,^[58]^ and they have been further analyzed following the procedure described in ref. [38]. The orientation of the surface with respect to the linear polarization (*s* and *p*) of the synchrotron beam was changed by rotating the sample around the beam axis while keeping the incident angle (6°) of the synchrotron light fixed.

The NEXAFS and XMCD spectra at the Fe l‐edge were measured at the X‐Treme beamline^[59]^ of the Swiss Light Source by recording the sample drain current in total electron yield mode. The X‐ray beam was impingent at normal (*θ*=0°) or grazing (*θ*=70°) incidence with respect to the sample surface, with the magnetic field collinear with the beam propagation direction. No spectral changes over time were observed, indicating the absence of beam damage. The temperature at the sample surface was 3 K.

The clean Cu(100) surface was prepared by a standard procedure: cycles of Ar^+^ ion sputtering at 2.0 keV followed by annealing at 800 K. FePc molecules (Sigma–Aldrich, ≥95 % purity) were thermally sublimated at 570 K from a home‐made Knudsen cell type evaporator onto the copper substrate kept at room temperature. As the monolayer coverage is long‐range ordered, the achievement of the desired coverage was monitored using reflective high‐energy electron diffraction (RHEED, at ALOISA) or low energy electron diffraction (LEED). The NO_2_ gas was introduced through a precision leak valve (partial pressure of 5×10^−7^ mbar) and its dosing took place in the preparation chamber while keeping the sample at room temperature. The oxygen‐covered Cu(100) surface, which shows a (√2×√2R)45° reconstruction confirmed by LEED pattern was prepared by exposing the Cu(100) surface to 800 L of O_2_ while keeping the sample temperature at 500 K.^[48]^

### Computational details

**Absorption spectra simulations**: Optimization calculations are performed by exploiting the Amsterdam Density‐Functional (ADF) software package.^[60]^ Numerical experiments have been carried out by running spin‐unrestricted, nonrelativistic DFT calculations with generalized gradient corrections self‐consistently included through the Becke–Perdew formula[^61^, ^62^] and by adopting a triple‐ζ with a polarization function Slater‐type basis set for all the atoms. MPc L_2,3_‐edges XA spectra[^39^, ^49^, ^63^, ^64^, ^65^] and Fe complexes[^66^, ^67^, ^68^] have been modelled by evaluating excitation energies and corresponding oscillator strengths (f) for transitions having the M 2p‐based MOs as initial spin orbitals through the use of the module ROCIS of the ORCA program package.^[69]^ Spectra have been simulated with the DFT/ROCIS method,^[70]^ which includes SOC in a molecular Russell‐Saunders fashion,^[70]^ by adopting the B3LYP exchange‐correlation (XC) functional^[72]^ and by using the def2‐TZVP(‐f) basis set.[^72^, ^73^] The combined use of DFT and configuration interaction needs a set of three semi‐empirical parameters (c1=0.21, c2=0.49, and c3=0.29).^[69]^ Moreover, the resolution of identity approximation has been used with the def‐TZVP/J basis set[^72^, ^73^] and the zeroth‐order regular approximation (ZORA) has been adopted to treat the scalar relativistic effects.^[74]^

**Photoemission tomography simulations**: Theoretical photoemission simulations were based on results obtained within the framework of DFT. The calculations of the gas phase FePc have been performed by the NWCHEM^[75]^ DFT code, using the Becke three‐parameter Lee–Yang–Parr (B3LYP)[^61^, ^76^, ^77^] hybrid. The simulated momentum maps of the gas‐phase FePc molecule were obtained as the FTs of the respective Kohn–Sham (KS) orbitals.^[31]^

## Conflict of interest

The authors declare no conflict of interest.

## Supporting information

As a service to our authors and readers, this journal provides supporting information supplied by the authors. Such materials are peer reviewed and may be re‐organized for online delivery, but are not copy‐edited or typeset. Technical support issues arising from supporting information (other than missing files) should be addressed to the authors.

Supplementary
