# Supplementary material for: Ferrous to Ferric Transition in Fe‐Phthalocyanine Driven by NO2 Exposure
Source: Chemistry. 2021 Jan 25;27(10):3526–35. doi: 10.1002/chem.202004932 (PMC7898877; doi:10.1002/chem.202004932)
Supplement: Supplementary file 1 — Supplementary [file CHEM-27-3526-s001.pdf]

Author Contributions

I.C., S.C., M.Ca. and V.F. drafted the manuscript with an essential contribution from G.Z. and L.F. P.P. performed the PT simulations. S.C. and M.Ca. performed the adsorption spectra simulations. Experiments have been performed by I.C., H.M.S., G.Z. and V.F. with the support of L.F., A.C., A.V., M.J. and C.P. Data analysis have been carried out by I.C. and V.F. All authors discussed the results and reviewed the manuscript.
